# Differential RNA Editing and Intron Splicing in Soybean Mitochondria during Nodulation

**DOI:** 10.3390/ijms21249378

**Published:** 2020-12-09

**Authors:** Yuzhe Sun, Min Xie, Zhou Xu, Koon Chuen Chan, Jia Yi Zhong, Kejing Fan, Johanna Wong-Bajracharya, Hon-Ming Lam, Boon Leong Lim

**Affiliations:** 1 School of Biological Sciences, University of Hong Kong, Pokfulam, Hong Kong, China; yzsun@connect.hku.hk (Y.S.); JodieXu85@hotmail.com (Z.X.); mikchankc@gmail.com (K.C.C.); zjiaer@gmail.com (J.Y.Z.); 2 Center for Soybean Research of the State Key Laboratory of Agrobiotechnology, The Chinese University of Hong Kong, Shatin, Hong Kong, China; feixue1039@gmail.com (M.X.); kejing68164614@gmail.com (K.F.); johannawh.wong@gmail.com (J.W.-B.); 3 School of Life Sciences, The Chinese University of Hong Kong, Shatin, Hong Kong, China

**Keywords:** complex I, intron splicing, maturase, NDH, RNA editing, mitochondria

## Abstract

Nitrogen fixation in soybean consumes a tremendous amount of energy, leading to substantial differences in energy metabolism and mitochondrial activities between nodules and uninoculated roots. While C-to-U RNA editing and intron splicing of mitochondrial transcripts are common in plant species, their roles in relation to nodule functions are still elusive. In this study, we performed RNA-seq to compare transcript profiles and RNA editing of mitochondrial genes in soybean nodules and roots. A total of 631 RNA editing sites were identified on mitochondrial transcripts, with 12% or 74 sites differentially edited among the transcripts isolated from nodules, stripped roots, and uninoculated roots. Eight out of these 74 differentially edited sites are located on the *matR* transcript, of which the degrees of RNA editing were the highest in the nodule sample. The degree of mitochondrial intron splicing was also examined. The splicing efficiencies of several introns in nodules and stripped roots were higher than in uninoculated roots. These include *nad1* introns 2/3/4, *nad4* intron 3, *nad5* introns 2/3, *cox2* intron 1, and *ccmFc* intron 1. A greater splicing efficiency of *nad4* intron 1, a higher NAD4 protein abundance, and a reduction in supercomplex I + III_2_ were also observed in nodules, although the causal relationship between these observations requires further investigation.

## 1. Introduction

Soybean is an important cash crop for protein and edible oil. Its ability to perform symbiotic nitrogen fixation in root nodules makes it a nitrogen-rich food source. Root cells obtain photo-assimilates from source tissues and catabolize these chemical compounds through root mitochondria to generate the ATPs required for various physiological and biochemical processes. In eukaryotic cells, cellular respiration mainly takes place inside mitochondria. The tricarboxylic acid (TCA) cycle and mitochondrial electron transport chain (mETC) convert biochemical energy from nutrients into ATP, which is an important energy currency of the cell.

The energy demand in root nodules is much higher than in uninoculated roots, since nitrogen fixation consumes a tremendous amount of energy [1]. Therefore, substantial differences in carbon metabolism and mitochondrial activities between root nodules and uninoculated root cells are expected. Sucrolytic activities (invertase and sucrose synthase) are found to be 3–4-fold higher in root nodules than in uninoculated roots and these enzymatic activities are restricted to the uninoculated cortical tissue and are absent in the infected central tissue of nodules [2]. Bacteroids of the nodules were shown to exhibit a limited capacity to utilize carbohydrates [3], with limited glycolytic activities and no sucrolytic activities. It is reported that sucrose taken up by root nodule cells is converted into malate and succinate as the main energy sources for bacteroids [1]. Hence, to facilitate the carbohydrate supply to bacteroids, the physiology of soybean mitochondria in nodules is significantly different from the uninoculated roots. Mitochondria in nodules were shown to oxidize malate at a rate 2-fold higher than cotyledon mitochondria. However, the activities of TCA cycle enzymes, except malate and succinate dehydrogenases, were lower in nodule mitochondria [4].

The vast majority of mitochondrial proteins from these crucial metabolic pathways are encoded in the nucleus and, after their translation in the cytoplasm, are imported into the mitochondria [5,6]. There are 110 predicted open reading frames in soybean mtDNA and 36 of them can be translated into proteins with known functions [7,8], including some subunits of the respiratory chain: NADH dehydrogenase (complex I), cytochrome oxidase (complex IV), ATP synthase (complex V), and cytochrome C biogenesis. Coordination of the expression and accumulation of mitochondrial proteins derived from the nuclear and mitochondrial genomes are complex, especially in the post-transcriptional regulation of mtDNA expression [9,10].

Some mitochondrial mRNA transcripts are subjected to C-to-U RNA editing, a house-keeping post-transcriptional process in plants [11]. RNA editing is carried out by the editosome complexes, which are composed of pentatricopeptide repeat (PPR) proteins, Multiple Organellar RNA Editing Factors (MORF), organelle RNA Recognition Motif-containing proteins (ORRM), and organelle zinc finger editing factor family (OZ) proteins [12]. RNA editing is complex and energy-consuming. The biological function and evolutionary significance of RNA editing in plants still remain unclear [13,14]. RNA editing may lead to amino acid substitutions and affect the function of the translated proteins [15]. Editing sites, which are edited with different efficiencies in different tissues, have also been identified in the plastids of diverse species [16,17].

RNA editing has been shown to be crucial for *nad1* splicing in *Oenothera* [18] and *nad7* splicing in maize [19]. The genomes of plant mitochondria house about 20 group-II introns and their splicing requires maturases [20,21,22]. In *Arabidopsis thaliana*, there is one maturase gene (*matR*) in the mitochondrial genome and four nuclear maturase genes (*nMat*1-4) in the nuclear genome [23]. The four nuclear-encoded maturases are imported into mitochondria after translation [24,25]. It has been experimentally demonstrated that nuclear-encoded maturases [20,23,26] and MatR [27] are required for the splicing of various group-II introns in Arabidopsis mitochondria and the assembly of functional complex I. Here, we compare the degrees of RNA editing and intron splicing on mitochondrial transcripts in soybean nodules and roots.

## 2. Results

### 2.1. RNA Sequencing and Differentially Expressed Mitochondrial Genes

To study the differences in mitochondrial transcripts between soybean nodules and uninoculated roots, we collected nodule (N) samples 28 days after rhizobium inoculation. Stripped roots (SR), main roots after the removal of nodules, and uninoculated roots (UR) of the same age were also harvested. We extracted total RNA from N, SR, and UR for RNA sequencing (RNA-seq) analysis (Table 1). The BioProject accession numbers of the RNA-seq data are PRJNA627909 (UR) and PRJNA626514 (N and SR), respectively. Here, we focused on mitochondrial gene expression and identified 93 gene transcripts (Supplementary Table S1). Besides transcripts encoding for hypothetical proteins, we identified 14 transcripts differentially expressed between N and UR, SR and UR, or SR and N (fold change > 1.5, *p*-value < 0.05 using Student’s *t*-test). These include transcripts of cytochrome c biogenesis C/F_N_ (*ccmC*, *ccmF_N_*), cytochrome c oxidase subunit III (*cox3*), NADH dehydrogenase subunit 1/2/4L/5 (*nad1*, *nad2*, *nad4L*, *nad5*), and some ribosomal proteins (Table 2). Compared to UR, eight transcripts, including six *nad* transcripts and two ribosomal protein transcripts, were significantly upregulated in SR, while the abundance of six transcripts including *cox3*, *nad1*, *nad5*, and three ribosomal protein transcripts was significantly higher in N. On the contrary, *ccmF_N_* was significantly down-regulated in N compared with UR. The abundance of four transcripts, including *cytochrome c biogenesis C/F_N_* (*ccmC*, *ccmF_N_*) and *ribosomal proteins L5* and *S14*, was lower in N than in SR, whereas the abundance of *cox3* and *rps12* transcripts was higher in N when compared with SR and UR.

### 2.2. Differential RNA Editing in Root Nodules

RNA-seq reads were mapped to the mitochondrial genome. A total of 631 RNA editing sites, with at least 15% edited reads in all three biological replicates of any one of the three samples, were identified (Supplementary Table S2). The average editing degrees of the three groups (N, SR, and UR) were compared, and 74 sites with ≥ 15% differences in the editing degrees between any two of the three tissues were identified (Supplementary Table S3). Out of these 74 editing sites, 12 sites were intronic, 23 sites were synonymous, and 39 sites were non-synonymous. There were a few observations: (i) UR showed higher editing degrees in *atp1-1*, *atp1-2*, *atp1-3*, and *atp1-4* than the other two samples, whereas all these sites are synonymous; (ii) There were 29 differentially edited sites in *nad* transcripts (*nad1*, *nad2*, *nad4*, *nad4L*, *nad5* and *nad7*). Most of these sites were intronic (11 sites) or synonymous (nine sites). Only six and three sites in *nad1* and *nad4*, respectively, could lead to amino acid substitutions; (iii) There were 18 differentially edited sites in ribosomal protein transcripts (*rpl5*, *rps1*, *rps4*, *rps10* and *rps12*). Only one site was intronic (*rps10*), but all the other sites could lead to amino acid substitutions. Comparing N to UR, 16 sites in *rps 1*, *4*, *10*, and *12* had higher editing degrees, while two sites in *rpl5* had lower editing degrees.

### 2.3. matR Transcripts Underwent Extensive RNA Editing in Root Nodules

**Figure 1:**
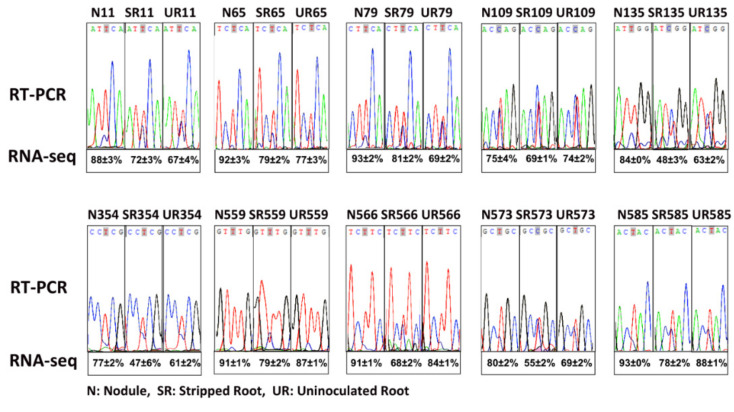
Validation of *matR* RNA editing by RT-PCR and Sanger sequencing. N, nodule, SR, stripped root, UR, uninoculated root. The numbers (e.g., N11) represented the editing site locates in the 11th amino acid codon of the *GlmaxMp73* (*matR*) transcript. The average percentages (with SD values) of RNA editing of three biological replicates obtained by RNA-seq are shown below the Sanger sequences.

We identified 17 editing sites on *matR*, of which eight sites had been identified in *A. thaliana* [15] (Table 3) and 11 sites had been reported previously in uninoculated soybean by comparing genomic DNA and cDNA sequences [28]. Out of these 17 editing sites, the degrees of RNA editing of eight sites were higher in the N than UR and SR, of which seven could lead to amino acid substitutions. To confirm that these sites were actually edited, RT-PCR and Sanger sequencing were performed on three biological replicates of each sample (Supplementary Figure S1). In general, the Sanger sequencing data confirmed the next-generation sequencing (NGS) data, and both showed that N had a higher degree of editing than UR and SR in multiple editing sites (Figure 1 and Table 2).

### 2.4. Identification of Nuclear-Encoded Mitochondrial Intron Maturases in the Soybean Genome

**Figure 2:**
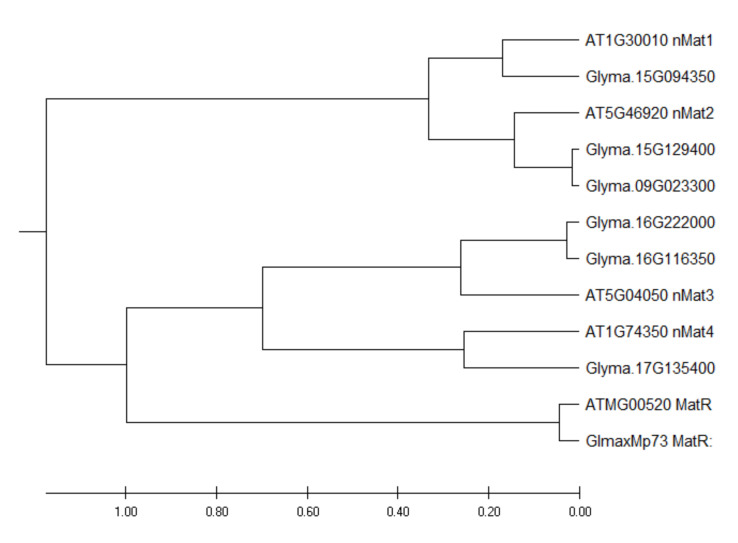
Phylogenetic tree of soybean intron maturases. Evolutionary analysis was conducted with MEGA X using the Unweighted Pair Group Method with Arithmetic Mean (UPGMA) method [29].

By using the protein sequences of the four Arabidopsis nuclear maturases as baits to search the soybean genome, six homologous nuclear maturases were identified. Among these transcripts, two are likely to have arisen from gene duplication (Figure 2). The expression levels of these six transcripts are presented in Supplementary Table S4. Among these six transcripts, only a transcript homologous to *AtnMAT4* had a significantly higher (2X) expression in N than in UR. By contrast, four transcripts homologous to *AtnMAT1-4* expressed significantly higher in SR than in UR (Supplementary Table S4).

### 2.5. Intron Splicing of Mitochondrial Transcripts

**Figure 3:**
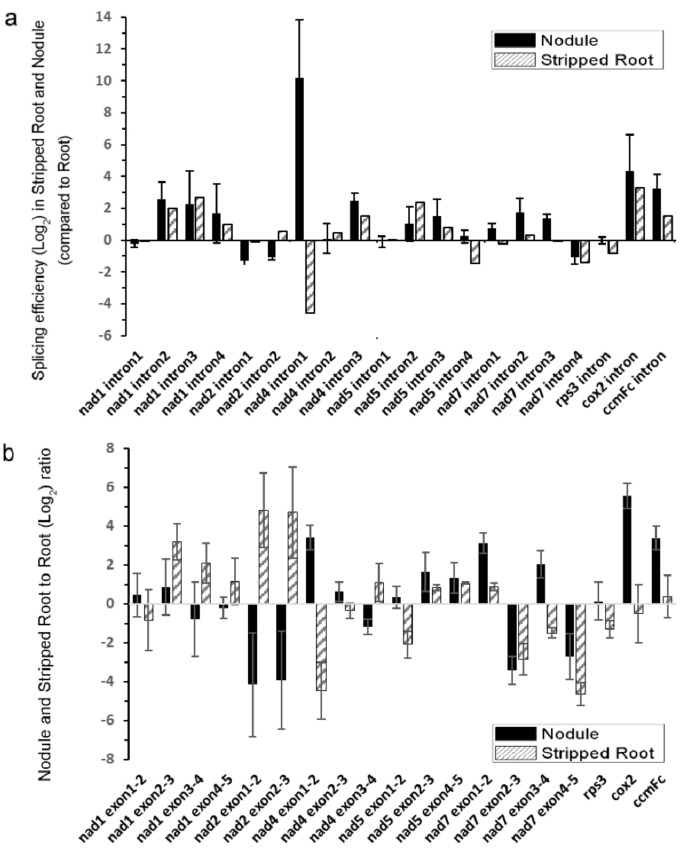
Quantitative RT-PCR analysis of splicing efficiency of mitochondrial transcripts. (**a**) Splicing efficiencies of mitochondrial transcripts in nodule and stripped root samples were compared with that of the root sample. The ratio of spliced mRNA to unspliced mRNA is designated as the splicing efficiency. The splicing efficiencies of nodules or stripped roots were divided by the splicing efficiency of roots. (**b**) The relative abundance of spliced mitochondrial mRNAs of nodule and stripped root samples were compared with that of roots. The histogram shows the log_2_ ratios of spliced transcripts. Values are means SD of three biological replicates.

Next, the splicing efficiencies of the 20 mitochondrial introns were analyzed among the samples by qRT-PCR (Figure 3). The splicing efficiencies of several introns were higher in both N and SR than UR, such as *nad1* introns 2/3/4, *nad4* intron 3, *nad5* introns 2/3, *cox2* intron 1, and *ccmFc* intron 1. While the nuclear intron maturases responsible for the splicing of some of these introns were identified in *A. thaliana* [20,23,26], the scenario in soybean is complicated by the presence of six nuclear intron maturases in the soybean genome (Supplementary Table S4). It should be noted that the splicing efficiency of the intron 1 of *nad4* was greatly enhanced in N than in UR and SR in the following order (N > UR > SR).

### 2.6. In-Gel Activity Assay and Western Blotting

**Figure 4:**
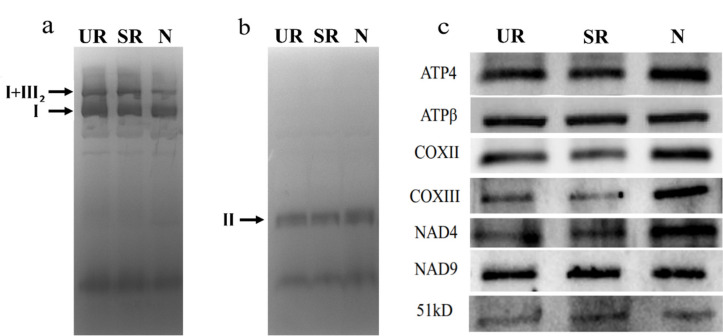
In-gel activity of the mitochondrial respiratory complexes separated by BN-PAGE. Complex I activity (**a**), Complex II activity (**b**), and Western blotting (**c**). UR, uninoculated roots; SR, stripped roots; N, nodules. I, complex I; I + III_2_, supercomplex composed of complex I and dimeric complex III; II, complex II. For Western blotting, 10 µg mitochondrial proteins were separated by SDS-PAGE and blotted with antibodies. Three biological replicates were run and the results were reproducible. The representative images are shown in (**a**–**c**).

Since qRT-PCR showed that there was more splicing of *nad4* intron 1 in N, we examined the abundance of NAD4 protein in our samples by Western blotting and in-gel activity assays (Figure 4). In-gel activity assay showed that the activity of protein complex II was similar among the three samples, and the complex I activity assay showed that while the activity of the monomeric complex I was similar among the three samples (Figure 4a,b), the activities of the supercomplex composed of complex I and dimeric complex III were lower in N (Figure 4a). In terms of protein abundance, while the level of NAD9 protein was similar among the three samples, a higher protein level of NAD4 was observed in the N sample in comparison to the other two samples. The N sample also had a higher protein abundance of COXIII, which could be due to a 2× increase in its mRNA abundance (Table 2).

## 3. Discussion

RNA editing is a common biological process in the plastids and mitochondria of land plants [30]. It is believed to be a post-transcriptional correction mechanism to circumvent mutations in the organellar genomes [11]. Since RNA editing is an energy-consuming and complex process, it must serve important biological functions, or otherwise it should not be maintained in higher plants during evolution. One possible advantage of RNA editing is to provide an additional regulatory mechanism to organelle physiology. RNA editing was shown to play important roles in organellar tRNA maturation [31] and intron splicing [18,19]. Several hundreds of RNA editing sites on mitochondrial transcripts and differential RNA editing were observed in our previous study on *A. thaliana* [15]. We showed that overexpression of AtPAP2, a phosphatase dually targets the outer membranes of chloroplasts and mitochondria [32,33] and plays a role in the import of pMORF3 into mitochondria [5], might influence cyt *c* biogenesis by modulating RNA editing of *ccm* transcripts through its interaction with the MORF proteins [15].

Here, the objective is to examine how nodule formation affects the degrees of RNA editing in soybean mitochondrial transcripts and investigate its possible biological purpose. We identified 631 RNA editing sites with at least 15% of edited transcripts in all three biological replicates of any one of the samples (Supplementary Table S2). Only 12% of these sites were differentially edited between any two of the three samples. One of the mitochondrial transcripts that underwent extensive RNA editing was the *matR* transcript (Table 3), which encodes an intron maturase that mediates group-II intron splicing. In a previous study, 510 RNA editing sites were identified in Arabidopsis mitochondrial transcripts and 124 sites were differentially edited between the wild-type and a high-energy, fast-growing, transgenic line [15]. There are 12 RNA editing sites in Arabidopsis *matR* transcripts, but none of them were differentially edited between the two lines [15]. Hence, the differential editing of *matR* transcripts is specific in our soybean samples and is likely to play a role in nodule function (Table 2). While plant mitochondria have lost most of their intron-encoded ORFs, a single intron maturase gene (*matR*) is retained in the fourth intron of *nad1* in seed plants [21] (Figure 2). In addition to the mitochondrial *matR* gene, phylogenetic analysis shows that the nuclear maturase genes are also conserved between *A. thaliana* and soybean, since the homologous sequences of all four Arabidopsis *nMat* homologs could be found in the soybean genome (Figure 2). Among the six soybean nuclear maturases, only a gene homologous to Arabidopsis *nMat4* exhibited a 2X higher expression in N than in UR (Supplementary Table S4).

By suppressing the expression of *matR* in Arabidopsis, the splicing efficiencies of *nad1* introns 3 and 4, *nad4* intron 3, *cox2* intron 1, and *ccmFc* intron 1 were downregulated, indicating that AtMatR carries out the splicing of these introns [27]. In soybean, the splicing efficiencies of these introns were higher in N and SR than in UR (Figure 3a). It should be noted that the abundance of *matR* transcripts were 27% and 38% higher in the SR and the N samples than in the UR sample (*p* < 0.005), respectively (Supplementary Table S1). Hence, the changes in the splicing efficiencies of these introns could be due to an increase in *matR* transcript abundance and/or due to the changes in RNA editing. The *nad4* intron 1 was not identified as a *matR*-mediated splicing site in Arabidopsis by *matR* knockdown experiments. However, it was identified in the MatR-ribonucleoprotein complex in an RNA co-immunoprecipitation experiment [27]. Hence, it is possible that RNA editing of *MatR* and the subsequent changes in its amino acid sequence might affect the composition of its associated ribonucleoprotein complex, thus affecting the splicing efficiency of *nad4* intron 1 by the other maturases. Alternatively, MatR might have an additional splicing activity toward *nad4* intron 1 in soybean. A P-type PPR protein, MISF68, was recently shown to be essential for the splicing of *nad4* intron 1 in *A. thaliana*, but the maturase responsible for its splicing has not yet been identified [34]. Nevertheless, several nuclear-encoded splicing factors can independently regulate the intron splicing in plant mitochondria, such as *nMat1-4*, mitochondrial transcription termination factors (*mTERF*s), and some PPR proteins. They are responsible for the splicing regulation of multiple introns in the mitochondrial genomes [35,36]. However, the most significant change between SR and N came from *nad4* intron 1 in the qRT-PCR results, suggesting that the splicing factor which targets the *nad4* intron 1 may play a role in nodule function.

Several *nad* genes are retained in the mitochondrial genome but are not transferred to the nucleus during evolution, probably because they encode the hydrophobic membrane subunits (NAD1-6) of the complex I [37]. Some *nad* introns are subjected to RNA editing (Supplementary Table S3). The latest research reveals that some RNA editing sites on the intronic sequences of maize mitochondrial *nad7* transcript can affect its intron splicing [19]. For example, in maize, it was previously shown that splicing would be abolished without the C-to-U editing at a specific position in two *nad7* introns [19]. In this study, 12 intronic RNA editing sites were identified, 11 of which were situated in *nad1/2/4/5/7* introns, including a site located in *nad4* intron 1 (base 102195, Supplementary Table S3). RNA editing of these intronic sequences could potentially affect intron splicing in soybean.

We observed the highest splicing efficiency of the *nad4* intron 1 in N compared to UR and SR (Figure 3), which might, in turn, affect the NAD4 protein abundance and supercomplex formation (Figure 4). In mammalian cells, spliced mRNA yielded more proteins than identical mRNA not made by splicing, possibly due to an enhanced association of spliced mRNA with polyribosomes [38]. This phenomenon is also observed in viruses [39]. Likewise, a higher splicing efficiency of the first intron of *nad4* in nodule (Figure 3a) might enhance the efficiency of translation. Evidentially, we detected a higher NAD4 protein abundance in nodule comparing to roots (Figure 4c) despite a similar *nad4* transcript abundance in all samples (Supplementary Table S1). NAD4 is a membrane component of complex I. In a maize *nad4* mutant (NCS2), while the missing NAD4 results in the destabilization of the NDH complex, a smaller complex can still exhibit complex I activity in the in-gel enzyme assay [40]. In BN-gel, complex I has two forms, either alone or forming a supercomplex (I + III_2_) with dimeric complex III, and their ratios are 40%/60% in plants, mammals, and fungi [41]. Our result showed that the mitochondria in nodules contain less I + III_2_ supercomplex, but contain a similar amount of free complex I to that of the root samples (Figure 4). Factors such as differential intron splicing, differential RNA editing of various *nad* transcripts and a change in NAD4 abundance could collectively affect the formation of the I + III_2_ supercomplex during nodulation. It was suggested that the I + III_2_ supercomplex may enable a more efficient electron transfer from complex I to complex III, as the close proximity of the two complexes enables efficient channeling of reduced quinol [42]. The lower abundance of supercomplex I + III_2_ in nodule mitochondria might affect electron transport rate in the mETC [41].

In nodules, nitrogen fixation by *S. fredii* is an extensive energy-consuming process. To fix one molecule of N_2_, 16 ATP molecules and eight electrons (and eight H^+^) are required [43]. C4-dicarboxylates (succinate, fumarate, and malate) were suggested to be the primary carbon source provided to the rhizobia by the root cells [1], and a recent study showed that malate, but not succinate or fumarate, is the essential dicarboxylate for bacteroid growth and symbiosis [44]. Normal root mitochondria do not exhibit substantial glycine decarboxylase (GDC) as there is no photorespiration in roots [45]. However, a proteomic study showed that nodule mitochondria contain a significantly higher amount of GDC, but a lower amount of ATP synthase α and β chains than uninoculated roots [46]. A high glycine flux is present in nodule mitochondria due to nitrogen fixation and ureide biosynthesis [47]. The presence of GDC in nodule mitochondria will generate a large amount of NADH. If the electron transport in the mETC is tuned down by the downregulation of the formation of supercomplex I + III_2_ [42], more NADH generated from GDC can be exported from the nodule mitochondria in the form of malate, which can then be consumed by the bacterioid for energy production. In this study, we reported the changes in RNA editing and intron splicing of the mitochondrial genome in soybean nodules. The relationship among RNA editing, intron splicing, and supercomplex I+III_2_ formation is complicated and requires further investigation.

## 4. Materials and Methods

### 4.1. Plant Materials and Sinorhizobium Fredii Inoculation

Nodules were harvested from a cultivated soybean C08 (*Glycine max*), which is closely related to Williams 82 from Illinois, USA [48]. Seeds of C08 were surface-sterilized with chlorine gas for 16 h and germinated in the dark in sterilized vermiculite with de-ionized water in the greenhouse. At three days post-germination, seedlings were transferred to individual containers with sterilized vermiculite with 1X low nitrogen nutrient solution (6.35 μM Ca(NO_3_)_2_, 133.59 μM CaSO_4_, 50.30 μM KCl, 12.17 μM MgSO_4_•7H_2_O, 39.04 μM K_2_HPO_4_, 15.31 μM ferric citrate, 2.31 μM H_3_BO_3_, 0.6 μM MnSO_4_, 0.07 μM ZnSO_4_, 0.16 μM CuSO_4_•5H_2_O, 0.01 μM H_2_MoO_4_) in a 16h/8h light/dark cycle at 25–30 °C [49] and inoculated with *S. fredii* strain *CCBAU45436* [50]. A set of uninoculated soybean control was also prepared alongside this. The *Rhizobium* strain was cultured on TY medium [51] at 28 °C with shaking at 180 rpm for 40 h. The cells were then pelleted and diluted in saline (0.9% *w/v* NaCl) to a final concentration of 10^20^ cells mL^−1^ (OD_600_ = 0.2) for inoculation. Ten-day-old seedlings were inoculated with 1 mL inoculum per plant. On the 28th day after inoculation, UR, N, and SR samples were collected separately and frozen immediately in liquid nitrogen and stored at −80 °C for RNA extraction. For mitochondria isolation, uninoculated roots, nodules, and the stripped roots were harvested and kept on ice for immediate isolation [5].

### 4.2. RNA Extraction and Sequencing

RNA was extracted using TRIzol (Life Technologies, Carlsbad, CA, USA) following the manufacturer’s protocol. Three biological replicates were prepared for each of the three conditions to produce a total of nine samples. Nine strand-specific RNA-seq libraries were generated using TruSeq RNA Sample Preparation Kit (Illumina, San Diego, CA, USA). Messenger RNA (mRNA) was enriched by depleting ribosomal protein RNA using Ribo-Zero Plant kit, rather than poly-A enrichment. These libraries were sequenced on Illumina Hiseq series platforms (sequencing service provided by Groken Bioscience, Hong Kong, China).

### 4.3. Bioinformatics Analysis

*G. max* Williams 82 v275 reference genome was downloaded from the Phytozome database (v9.0: https://phytozome.jgi.doe.gov/). The complete soybean mitochondrion genome was downloaded from the NCBI database (ID: NC_020455.1) [8]. The sequences were combined into one reference genome for subsequent analysis. Genomic variations (SNPs and Indels) between C08 and the reference genome were identified using the GATK pipeline (version 4.0.5.2) [52] according to GATK best practice workflow for germline short variant discovery (https://software.broadinstitute.org/gatk/best-practices/workflow?id=11145). Briefly, C08 DNA sequencing reads were mapped to the reference genome using BWA-MEM (version 0.7.15) implementation with default parameters. Duplicated reads were marked with GATK MarkDuplicates implementation. Then, base quality score recalibration was performed with known variation sites downloaded from the Phytozome database (v12). Then, SNPs and Indels between C08 and the reference genome were called based on mapped and quality score recalibrated reads using GATK HaplotypeCaller implementation.

RNA-seq reads were mapped to reference genome using Tophat2 (version 2.1.1) [53]. Properly mapped read pairs were assigned to each annotated gene by featureCounts from the subread package [54]. Read pair count of each mitochondrial gene was normalized by transcript length and total read count to calculate FPKM value.

To identify RNA editing sites, RNA-seq reads that could be mapped to the mitochondrial genome were extracted. SNPs were identified, and allele frequency was calculated for each polymorphic site using samtools mpileup (version 1.7) [55] and varScan2 (version 2.4.3) [56]. SNPs that were also identified by GATK with DNA reads were assumed to be germline variations and filtered. The remaining SNPs were considered as candidate RNA-editing sites.

### 4.4. RT-PCR and Quantitative RT-PCR

cDNAs were synthesized with M-MLV RT (200 U/μL) (Invitrogen, Hong Kong, China) and random hexamers (Invitrogen, Hong Kong, China), according to the manufacturer’s instructions. To verify the differential editing of mitochondrial *matR* transcript observed in RNA-seq data, specific primers were designed to amplify regions that contain each *matR* editing site by RT-PCR (Supplementary Table S5). PCR products were sent out to BGI-Shenzhen for Sanger sequencing.

Quantitative reverse transcription PCR (qRT-PCR) analysis was carried out using the same batch of RNA samples. Primers used in qRT-PCR were derived from a previous *A. thaliana* study in which specific oligonucleotides were designed to target intron-exon and exon-exon regions [23]. New primers were designed based on the homology between *A. thaliana* and soybean mitochondrial genomes (Supplementary Table S5). SYBR Green Master Mix (ABIsystems, Hong Kong) was used in a 10 μL volume PCR reactions. Tubulin gene (Gene ID: Glyma20g27280) was used as the internal house-keeping control. The assessment of relative expression levels was calculated using the Ct comparative threshold method [15,57]. The expression levels of spliced mRNA and unspliced mRNA were first calculated and the ratio was defined as the splicing efficiency. To compare the splicing efficiencies between samples, the splicing efficiencies of SR and N were divided by that of UR (Figure 3a).

### 4.5. Isolation of Soybean Mitochondria

Soybean mitochondria were isolated as previously described with modifications [58,59]. All procedures were done at 4 °C including sample harvest and centrifugation. A total of 20 g of the UR, 20 g of SR, and 10 g of the N were sampled and ground in 50 mL grinding buffer (pH 7.5) containing 0.3 M sucrose, 25 mM Tetrasodiumpyrophosphate, 2 mM EDTA, 10 mM KH_2_PO_4_, 1.0% (*w/v*) PVP-40, 1% (*w/v*) BSA, 20 mM ascorbate and L-cysteine. After 2 min of grinding, the homogenates were filtered through a double layer of Miracloth and rinsed again with 50 mL grinding buffer and centrifuged at 4000 *g* for 5 min. The supernatant was transferred to a tube and centrifuged at 10,000 *g* for 15 min. The pellet was resuspended in a wash buffer (0.3 M sucrose, 10 mM TES, 0.1% (*w/v*) BSA, pH 7.5) and layered on 30 mL of wash buffer containing 45% (*v/v*) Percoll in a tube and centrifuged at 40,000 *g* for 30 min. The crude mitochondria located in a tight brown band near the top of the tube were transferred and diluted at least 5-fold with the wash buffer and concentrated by centrifuging at 15,000 *g* for 10 min. The pellet was resuspended in around 5 mL of wash buffer before loading to a continuous gradient solution containing 0 to 4.4% (*v/v*) PVP-40 and 28% (*v/v*) Percoll in the wash buffer. After centrifugation at 40,000 *g* for 30 min, the mitochondria were concentrated in a pale-yellow band located near the bottom of the tube. This layer was then transferred to a new polycarbonate centrifuge tube with the BSA-free wash buffer and centrifuged at 2450 g for 15 min. After 3-4 wash steps, the mitochondria pellet was resuspended in the wash buffer without BSA. After the protein concentration was determined by the Bradford protein assay (BIO-RAD, Hercules, CA, USA), the mitochondria were stored in aliquots at −80 °C.

### 4.6. Blue Native-Polyacrylamide Gel Electrophoresis (BN-PAGE) and In-Gel Enzyme Activity Staining

The mitochondrial protein complex extraction and BN-PAGE were carried out as previously published with modifications [60]. An equal amount of mitochondria was collected by centrifugation at 14,300 *g* for 10 min at 4 °C and resuspended in 5% (*w/v*) digitonin extraction buffer to a final ratio of 10:1 (*w/v*) of protein to detergent and incubated on ice for 20 min. The solubilized proteins were then centrifuged at 18,300 *g* for 20 min at 4 °C. The supernatant (100 µg per sample) was transferred to a new tube supplemented with 5% (*v/v*) Serva blue G250 solution by a final ratio of 100:1 (*w/v*) of protein to dye and was loaded to a standard 1.0 mm × 10 well NativePAGE^TM^ 3–12% Bis-Tris Gel (Invitrogen, Hong Kong, China). The cathode buffer (50 mM Tricine, 15 mM bis-Tris, 0.02% (*w/v*) Serva Blue G250, pH 7.0) and the anode buffer (50 mM Bis-Tris, pH 7.0 with HCl) were freshly prepared. The electrophoresis was carried out at 4 °C at 75 V for 30 min, followed by 100 V for 30 min, 125 V for 30 min, 150 V for 1 h, 175 V for 30 min, and then set to a constant voltage 200 V until the sample reached the bottom of the gel.

After electrophoresis, the gels were washed twice with MiliQ water for 10 min. Then, the gels were equilibrated in the appropriate reaction buffer without reagents for 10 min. The gel was then incubated in a fresh reaction buffer of complex I (0.1 M Tris, 0.2 mM NADH, 0.2% (*w/v*) nitro-blue tetrazolium, pH 7.4) for 30 min. The other gel was incubated in a fresh reaction buffer of complex II (50 mM KH_2_PO_4_, 0.1 mM ATP, 0.2 mM Phenazine methosulphate, 10 mM succinate, 0.2% (*w/v*) nitro-blue tetrazolium) for 2 h. The reactions were terminated by fixing the gels in 40% (*v/v*) methanol and 10% (*v/v*) acetic acid for at least 1 h. The gels were destained overnight in 20% (*v/v*) methanol to remove residual Serva Blue G. For Western blot analysis, 10 µg mitochondrial proteins were run into SDS-PAGE and the proteins were transferred to Hybond-P nitrocellulose membranes (GE Healthcare, Hong Kong, China). The following antibodies were used: anti-beta subunit of ATP synthase (ATP4, PhytoAB PHY0587S, 1:1000; ATPβ, Agrisera AS05 085, 1:4000); anti-cytochrome oxidase subunit II (COXII, Agrisera AS04 053A, 1:2000); anti-cytochrome oxidase subunit III (COXIII, PhytoAB PHY0580S, 1:1000); anti-NAD4 (PhytoAB PHY0511S, 1:1000); anti-NAD9 (from Dr. G. Bonnard, 1:50,000); anti-51kDa (PhytoAB PHY0525S, 1:1000). The signals were developed by the Enhanced Chemiluminescence method (ECL; GE Healthcare, Hong Kong, China).

## Figures and Tables

**Table 1:** Summary of RNA-seq sequencing data.

Sample		Read Length (bp)	Read Count	Total Length (bp)
Stripped root	D3	125	66,851,390	8,356,423,750
	E3	125	75,008,428	9,376,053,500
	F3	125	70,072,218	8,759,027,250
Nodule	D4	125	68,175,300	8,521,912,500
	E4	125	63,858,336	7,982,292,000
	F4	125	62,615,136	7,826,892,000
Control Root	C08-Root1	150	111,230,354	16,684,553,100
	C08-Root2	150	106,852,728	16,027,909,200
	C08-Root3	150	115,102,338	17,265,350,700

**Table 2:** Differentially expressed mitochondrial transcripts.

Gene ID	Gene	Function Description	UR FPKM	SR FPKM	N FPKM	N/UR	*p*-Value	N/SR	*p*-Value	SR/UR	*p*-Value
GlmaxMp01	*ccmC*	cytochrome c biogenesis C	15,863	20,620	10,842	0.68	0.000	**0.53**	0.000	1.30	0.001
GlmaxMp51	*ccmFn*	cytochrome c biogenesis FN	4048	2982	1586	**0.39**	0.000	**0.53**	0.000	0.74	0.006
GlmaxMp04	*cox3*	cytochrome c oxidase subunit III	31,643	31,486	60,520	**1.91**	0.000	**1.92**	0.000	1.00	0.943
GlmaxMp20-2	*nad1*	NADH dehydrogenase subunit 1	12,430	21,952	27,133	**2.18**	0.000	1.24	0.002	**1.77**	0.000
GlmaxMp31-1	*nad2*	NADH dehydrogenase subunit 2	12,565	19,324	16,626	1.32	0.048	0.86	0.001	**1.54**	0.009
GlmaxMp32	*nad4L-1*	NADH dehydrogenase subunit 4L	30,659	51,843	39,578	1.29	0.257	0.76	0.007	**1.69**	0.035
GlmaxMp46	*nad4L-2*	NADH dehydrogenase subunit 4L	29,685	47,502	36,863	1.24	0.353	0.78	0.009	**1.60**	0.057
GlmaxMp05-1	*nad5*	NADH dehydrogenase subunit 5	17,739	26,994	25,170	1.42	0.025	0.93	0.036	**1.52**	0.014
GlmaxMp05-2	*nad5*	NADH dehydrogenase subunit 5	26,303	42,023	55,654	**2.12**	0.000	1.32	0.003	**1.60**	0.004
GlmaxMp07	*rp15*	ribosomal protein subunit L5	15,181	19,217	10,240	0.67	0.145	**0.53**	0.000	1.27	0.213
GlmaxMp53	*rps1*	ribosomal protein S1	9695	21,173	17,874	**1.84**	0.013	0.84	0.005	**2.18**	0.004
GlmaxMp16	*rps12*	ribosomal protein S12	10,421	11,110	19,813	**1.90**	0.001	**1.78**	0.000	1.07	0.572
GlmaxMp08	*rps14*	ribosomal protein subunit S14	24,486	25,459	14,704	0.60	0.077	**0.58**	0.000	1.04	0.826
GlmaxMp52	*rps4*	ribosomal protein S4	6737	15,174	16,296	**2.42**	0.004	1.07	0.012	**2.25**	0.006

Three samples of each tissue were sequenced and the average FPKM is presented in this table. Student’s t-tests were carried out between tissues. Ratios that are significantly increased or decreased (fold change > 1.5 or < 0.067, *p*-value < 0.05) are shown in bold.

**Table 3:** RNA editing sites in *matR* transcripts.

Genome Position	Transcript Position	A.A. Position	Codon Change	A.A. Change	Nodule Average Editing Degree (%)	Stripped Root Average Editing Degree (%)	Root Average Editing Degree (%)	Changes (N-UR) (%)	Changes (N-SR) (%)	Changes (SR-UR) (%)	Editing Sites in Arabidopsis (Y/N) *	Editing Degree (%) *	Editing Sites in Soybean (Y/N) #
340,005	32	11	TCC>-<TTC	S>-<F	88 ± 3 ^a^	72 ± 3 ^b^	67 ± 4 ^b^	21	16	5	No	-	Yes
339,890	147	49	TTC>-<TTT	No (F)	26 ± 1 ^a^	12 ± 2 ^b^	13 ± 4 ^b^	13	14	−1	No	-	No
339,844	193	65	CCA>-<TCA	P>-<S	92 ± 3 ^a^	79 ± 2 ^b^	77 ± 3 ^b^	15	13	2	No	-	Yes
339,801	236	79	TCC>-<TTT	S>-<F	93 ± 2 ^a^	81 ± 2 ^b^	69 ± 2 ^c^	24	12	12	No	-	Yes
339,711	326	109	CCA>-<CTA	P>-<L	75 ± 4 ^a^	69 ± 1 ^b^	74 ± 2 ^a^	0	6	−6	Yes	87	Yes
339,633	404	135	TCG>-<TTG	S>-<L	84 ± 0 ^a^	48 ± 3 ^b^	63 ± 2 ^c^	20	35	−15	Yes	90	No
339,126	911	304	GCC>-<GTC	A>-<V	17 ± 2 ^a^	13 ± 0 ^b^	19 ± 5 ^ab^	−2	4	−6	No	-	No
338,976	1061	354	CCC>-<CTC	P>-<	77 ± 2 ^a^	47 ± 6 ^b^	61 ± 2 ^c^	15	30	−15	No	-	Yes
338,495	1542	514	CCC>-<CCT	No (P)	42 ± 1 ^a^	22 ± 2 ^b^	20 ± 4 ^b^	22	20	2	Yes	43	No
338,361	1676	559	TCT>-<TTT	S>-<F	91 ± 1 ^a^	79 ± 2 ^b^	87 ± 1 ^c^	4	13	−9	Yes	88	Yes
338,340	1697	566	CCT>-<CTT	P>-<L	91 ± 1 ^a^	68 ± 2 ^b^	84 ± 1 ^c^	7	23	−16	Yes	84	Yes
338,320	1717	573	CGC>-<TGT	R>-<C	80 ± 2 ^a^	55 ± 2 ^b^	69 ± 2 ^c^	11	25	−14	Yes	83	Yes
338,306	1731	577	TAC>-<TAT	No (Y)	97 ± 1 ^a^	93 ± 1 ^b^	95 ± 0 ^c^	2	4	−2	No	-	No
338,284	1753	585	CAC>-<TAC	H>-<Y	93 ± 0 ^a^	78 ± 2 ^b^	88 ± 1 ^c^	5	15	−10	Yes	79	Yes
338,214	1823	608	CCC>-<CTC	P>-<L	83 ± 1 ^a^	72 ± 1 ^b^	71 ± 1 ^b^	12	11	2	No	-	Yes
338,196	1841	614	TCA>-<TTA	S>-<L	97 ± 1 ^a^	91 ± 1 ^b^	96 ± 1 ^a^	1	7	−5	Yes	93	Yes
338,186	1851	617	GTC>-<GTT	No (V)	42 ± 2 ^a^	33 ± 1 ^b^	39 ± 2^a^	3	9	−6	No	-	No

The *MatR* gene is located on the negative strand. All the RNA editing sites are C-< U changes. Sites validated by RT-PCR in Figure 1 were underlined. * Editing sites identified in *Arabidopsis thaliana* (Sun et al., 2017). # Editing sites previously reported in soybean *matR* (Thomson et al., 1994). ^abc^ Sites with significant differences (Student *t*-test, *p* < 0.05) in the degree of editing between the samples were labelled by different letters.

## Supplementary Materials

The following are available online at https://www.mdpi.com/1422-0067/21/24/9378/s1. Figure S1: Sanger sequencing of three biological replicates of each sample. Table S1: Normalized read count for each mitochondrial transcript in the three tissues. Table S2. RNA edited sites in the mitochondrial genome identified in this study. Table S3. Differentially edited RNA editing sites. Table S4: Expression of nuclear intron maturases in the three tissues. Table S5. Primers used in this study.

## References

[1] Liu A., Contador C., Fan K., Lam H.-M. (2018). Interaction and regulation of carbon, nitrogen, and phosphorus metabolisms in root nodules of legumes. Front. Plant Sci..

[2] Copeland L., Vella J., Hong Z. (1989). Enzymes of carbohydrate metabolism in soybean nodules. Phytochemistry.

[3] Dunn M.F. (1998). Tricarboxylic acid cycle and anaplerotic enzymes in rhizobia. FEMS Microbiol. Rev..

[4] Bryce J.H., Day D.A. (1990). Tricarboxylic acid cycle activity in mitochondria from soybean nodules and cotyledons. J. Exp. Bot..

[5] Law Y.S., Zhang R., Guan X., Cheng S., Sun F., Duncan O., Murcha M., Whelan J., Lim B.L. (2015). Phosphorylation and dephosphorylation of the presequence of pMORF3 during import into mitochondria from *Arabidopsis thaliana*. Plant Physiol..

[6] Law Y.S., Ngan L., Yan J., Kwok L.Y., Sun Y., Cheng S., Schwenkert S., Lim B.L. (2018). Multiple kinases can phosphorylate the N-terminal sequences of mitochondrial proteins in *Arabidopsis thaliana*. Front. Plant Sci..

[7] Mower J.P. (2020). Variation in protein gene and intron content among land plant mitogenomes. Mitochondrion.

[8] Chang S., Wang Y., Lu J., Gai J., Li J., Chu P., Guan R., Zhao T. (2013). The mitochondrial genome of soybean reveals complex genome structures and gene evolution at intercellular and phylogenetic levels. PLoS ONE.

[9] Takenaka M., Verbitskiy D., Merwe J.A.V.D., Zehrmann A., Brennicke A. (2008). The process of RNA editing in plant mitochondria. Mitochondrion.

[10] Giegé P., Sweetlove L.J., Cognat V., Leaver C.J. (2005). Coordination of nuclear and mitochondrial genome expression during mitochondrial biogenesis in Arabidopsis. Plant Cell.

[11] Ichinose M., Sugita M. (2016). RNA editing and its molecular mechanism in plant organelles. Genes.

[12] Sun T., Shi X., Friso G., Wijk K.V., Bentolila S., Hanson M.R. (2015). A zinc ginger motif-containing protein is essential for chloroplast RNA editing. PLoS Genet..

[13] Shikanai T. (2015). RNA editing in plants: Machinery and flexibility of site recognition. Biochim. Biophys. Acta BBA Bioenerg..

[14] Castandet B., Araya A. (2011). RNA editing in plant organelles. Why make it easy?. Biochem. Mosc..

[15] Sun Y., Law Y.S., Cheng S., Lim B.L. (2017). RNA editing of cytochrome c maturation transcripts is responsive to the energy status of leaf cells in *Arabidopsis thaliana*. Mitochondrion.

[16] Tseng C.-C., Lee C.-J., Chung Y.-T., Sung T.-Y., Hsieh M.-H. (2013). Differential regulation of Arabidopsis plastid gene expression and RNA editing in non-photosynthetic tissues. Plant Mol. Biol..

[17] Miyata Y., Sugita M. (2004). Tissue- and stage-specific RNA editing of *rps14* transcripts in moss (*Physcomitrella patens*) chloroplasts. J. Plant Physiol..

[18] B#xF6;rner G.V., Mörl M., Wissinger B., Brennicke A., Schmelzer C. (1995). RNA editing of a group II intron in Oenothera as a prerequisite for splicing. Mol. Gen. Genet..

[19] Xu C., Song S., Yang Y., Lu F., Zhang M., Sun F., Jia R., Song R., Tan B.-C. (2020). DEK46 performs C-to-U editing of a specific site in mitochondrial *nad7* introns that is critical for intron splicing and seed development in maize. Plant J..

[20] Keren I., Bezawork-Geleta A., Kolton M., Maayan I., Belausov E., Levy M., Mett A., Gidoni D., Shaya F., Ostersetzer-Biran O. (2009). AtnMat2, a nuclear-encoded maturase required for splicing of group-II introns in Arabidopsis mitochondria. RNA.

[21] Guo W., Mower J.P. (2013). Evolution of plant mitochondrial intron-encoded maturases: Frequent lineage-specific loss and recurrent intracellular transfer to the nucleus. J. Mol. Evol..

[22] Brown G.G., Francs-Small C.C.D., Ostersetzer-Biran O. (2014). Group II intron splicing factors in plant mitochondria. Front. Plant Sci..

[23] Keren I., Tal L., Francs-Small C.C.D., Araújo W.L., Shevtsov S., Shaya F., Fernie A.R., Small I., Ostersetzer-Biran O. (2012). nMAT1, a nuclear-encoded maturase involved in the trans-splicing of *nad1* intron 1, is essential for mitochondrial complex I assembly and function. Plant J..

[24] Bonen L. (2008). Cis- and trans-splicing of group II introns in plant mitochondria. Mitochondrion.

[25] Verbitskiy D., Härtel B., Zehrmann A., Brennicke A., Takenaka M. (2011). The DYW-E-PPR protein MEF14 is required for RNA editing at site *matR* -1895 in mitochondria of *Arabidopsis thaliana*. FEBS Lett..

[26] Cohen S., Zmudjak M., Francs-Small C.C.d., Malik S., Shaya F., Keren I., Belausov E., Many Y., Brown G.G., Small I. (2014). nMAT4, a maturase factor required for *nad1* pre-mRNA processing and maturation, is essential for holocomplex I biogenesis in Arabidopsis mitochondria. Plant J..

[27] Sultan L.D., Mileshina D., Grewe F., Rolle K., Abudraham S., G#x142;odowicz P., Niazi A.K., Keren I., Shevtsov S., Klipcan L. (2016). The reverse transcriptase/RNA maturase protein MatR is required for the splicing of various Group II introns in Brassicaceae mitochondria. Plant Cell.

[28] Thomson M.C., Macfarlane J.L., Beagley C.T., Wolstenholme D.R. (1994). RNA editing of *matR* transcripts in maize and soybean increases similarity of the encoded protein to fungal and bryophyte group II intron maturases: Evidence that *matR* encodes a functional protein. Nucleic Acids Res..

[29] Kumar S., Stecher G., Li M., Knyaz C., Tamura K. (2018). MEGA X: Molecular Evolutionary Genetics Analysis across computing platforms. Mol. Biol. Evol..

[30] Small I., Schallenberg-Rüdinger M., Takenaka M., Mireau H., Ostersetzer-Biran O. (2019). Plant organellar RNA editing: What 30 years of research has revealed. Plant J..

[31] Mareéchal-Drouard L., Ramamonjisoa D., Cosset A., Weil J., Dietrich A. (1993). Editing corrects mispairing in the acceptor stem of bean and potato mitochondrial phenylalanine transfer RNAs. Nucleic Acids Res..

[32] Sun F., Carrie C., Law S., Murcha M.W., Zhang R., Law Y.S., Suen P.K., Whelan J., Lim B.L. (2012). AtPAP2 is a tail-anchored protein in the outer membrane of chloroplasts and mitochondria. Plant Signal. Behav..

[33] Sun F., Suen P.K., Zhang Y., Liang C., Carrie C., Whelan J., Ward J.L., Hawkins N.D., Jiang L., Lim B.L. (2012). A dual-targeted purple acid phosphatase in *Arabidopsis thaliana* moderates carbon metabolism and its overexpression leads to faster plant growth and higher seed yield. New Phytol..

[34] Wang C., Aubé F., Quadrado M., Dargel-Graffin C., Mireau H. (2018). Three new pentatricopeptide repeat proteins facilitate the splicing of mitochondrial transcripts and complex I biogenesis in Arabidopsis. J. Exp. Bot..

[35] Hsu-Liang H., Wang H.-J., Hsieh M.-H., Hsieh H.-L., Jauh G.-Y. (2014). Arabidopsis mTERF15 is required for mitochondrial *nad2* intron 3 splicing and functional complex I activity. PLoS ONE.

[36] Zhao P., Wang F., Li N., Shi D.-Q., Yang W.-C. (2020). Pentatricopeptide repeat protein MID1 modulates *nad2* intron 1 splicing and Arabidopsis development. Sci. Rep..

[37] Braun H.-P., Binder S., Brennicke A., Eubel H., Fernie A.R., Finkemeier I., Klodmann J., König A.-C., Kühn >K., Meyer E. (2014). The life of plant mitochondrial complex I. Mitochondrion.

[38] Long R. (2004). Faculty Opinions recommendation of Splicing enhances translation in mammalian cells: An additional function of the exon junction complex. Genes Dev..

[39] Machinaga A., Ishihara S., Shirai A., Takase-Yoden S. (2016). Splicing of friend murine leukemia virus env-mRNA enhances its ability to form polysomes. Front. Microbiol..

[40] Karpova O.V., Newton K.J. (1999). A partially assembled complex I in NAD4-deficient mitochondria of maize. Plant J..

[41] Davies K.M., Blum T.B., Kuhlbrandt W. (2018). Conserved in situ arrangement of complex I and III_2_ in mitochondrial respiratory chain supercomplexes of mammals, yeast, and plants. Proc. Natl. Acad. Sci. USA.

[42] Hunte C. (2005). Faculty Opinions recommendation of structure of a mitochondrial supercomplex formed by respiratory-chain complexes I and III. Proc. Natl. Acad. Sci. USA.

[43] Seefeldt L.C., Hoffman B.M., Dean D.R. (2009). Mechanism of Mo-dependent nitrogenase. Annu. Rev. Biochem..

[44] Mitsch M.J., DiCenzo G.C., Cowie A., Finan T.M. (2017). Succinate transport is not essential for symbiotic nitrogen fixation by *Sinorhizobium meliloti* or *Rhizobium leguminosarum*. Appl. Environ. Microbiol..

[45] Gardeström P., Bergman A., Ericson I. (1980). Oxidation of glycine via the respiratory chain in mitochondria prepared from different parts of Spinach. Plant Physiol..

[46] Hoa L.T.-P., Nomura M., Kajiwara H., Day D.A., Tajima S. (2004). Proteomic analysis on symbiotic differentiation of mitochondria in soybean nodules. Plant Cell Physiol..

[47] Tajima S. (2004). Ureide biosynthesis in legume nodules. Front. Biosci..

[48] Qi X., Li M.-W., Xie M., Liu X., Ni M., Shao G., Song C., Yim A.K.-Y., Tao Y., Wong F.-L. (2014). Identification of a novel salt tolerance gene in wild soybean by whole-genome sequencing. Nat. Commun..

[49] Callow J.A., Vincent J.M. (1971). A manual for the practical study of root-nodule bacteria. J. Appl. Ecol..

[50] Rehman H.M., Cheung W.-L., Wong K.-S., Xie M., Luk C.-Y., Wong F.-L., Li M.-W., Tsai S.-N., To W.-T., Chan L.-Y. (2019). High-throughput mass spectrometric analysis of the whole proteome and secretome from *Sinorhizobium fredii* strains CCBAU25509 and CCBAU45436. Front. Microbiol..

[51] Beringer J.E. (1974). R factor transfer in *Rhizobium leguminosarum*. J. Gen. Microbiol..

[52] Auwera G.A.V.D., Carneiro M.O., Hartl C., Poplin R., Del Angel G., Levy-Moonshine A., Jordan T., Shakir K., Roazen D., Thibault J. (2013). From FastQ data to high-confidence variant calls: The genome analysis toolkit best practices pipeline. Curr. Protoc. Bioinform..

[53] Kim D., Pertea G., Trapnell C., Pimentel H., Kelley R., Salzberg S.L. (2013). TopHat2: Accurate alignment of transcriptomes in the presence of insertions, deletions and gene fusions. Genome Biol..

[54] Liao Y., Smyth G.K., Shi W. (2013). Feature Counts: An efficient general purpose program for assigning sequence reads to genomic features. Bioinformatics.

[55] Li H. (2011). A statistical framework for SNP calling, mutation discovery, association mapping and population genetical parameter estimation from sequencing data. Bioinformatics.

[56] Koboldt D.C., Zhang Q., Larson D.E., Shen D., McLellan M.D., Lin L., Miller C.A., Mardis E.R., Ding L., Wilson R.K. (2012). VarScan 2: Somatic mutation and copy number alteration discovery in cancer by exome sequencing. Genome Res..

[57] Liang C., Zhang Y., Cheng S., Osorio S., Sun Y., Fernie A.R., Cheung C.Y.M., Lim B.L. (2015). Impacts of high ATP supply from chloroplasts and mitochondria on the leaf metabolism of *Arabidopsis thaliana*. Front. Plant Sci..

[58] Day D., Neuburger M., Douce R. (1985). Biochemical characterization of chlorophyll-free mitochondria from pea leaves. Funct. Plant Biol..

[59] Lister R., Carrie C., Duncan O., Ho L.H., Howell K.A., Murcha M.W., Whelan J. (2007). Functional definition of outer membrane proteins involved in preprotein import into mitochondria. Plant Cell.

[60] Eubel H., Braun H.-P., Millar A.H. (2005). Blue-native PAGE in plants: A tool in analysis of protein-protein interactions. Plant Methods.

