# Supplementary material for: Differential RNA Editing and Intron Splicing in Soybean Mitochondria during Nodulation
Source: Int J Mol Sci. 2020 Dec 9;21(24):9378. doi: 10.3390/ijms21249378 (PMC7764374; doi:10.3390/ijms21249378)
Supplement: Supplementary file 1 [file ijms-21-09378-s001.zip › Supplementary Figure S1.pdf]

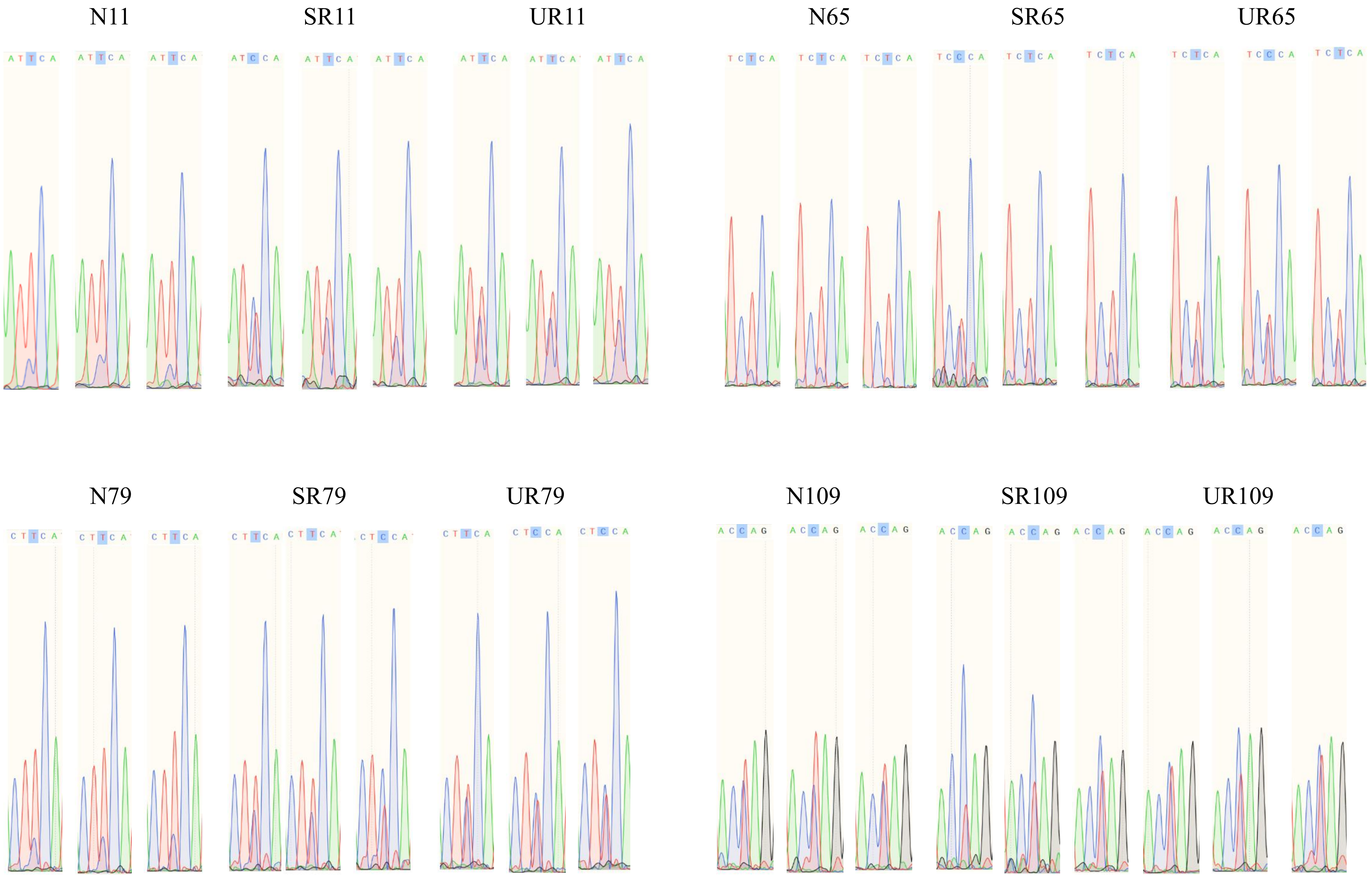

Supplementary Figure S1. Sanger sequencing results of three biological replicates of RNA editing sites on *matR* transcripts.

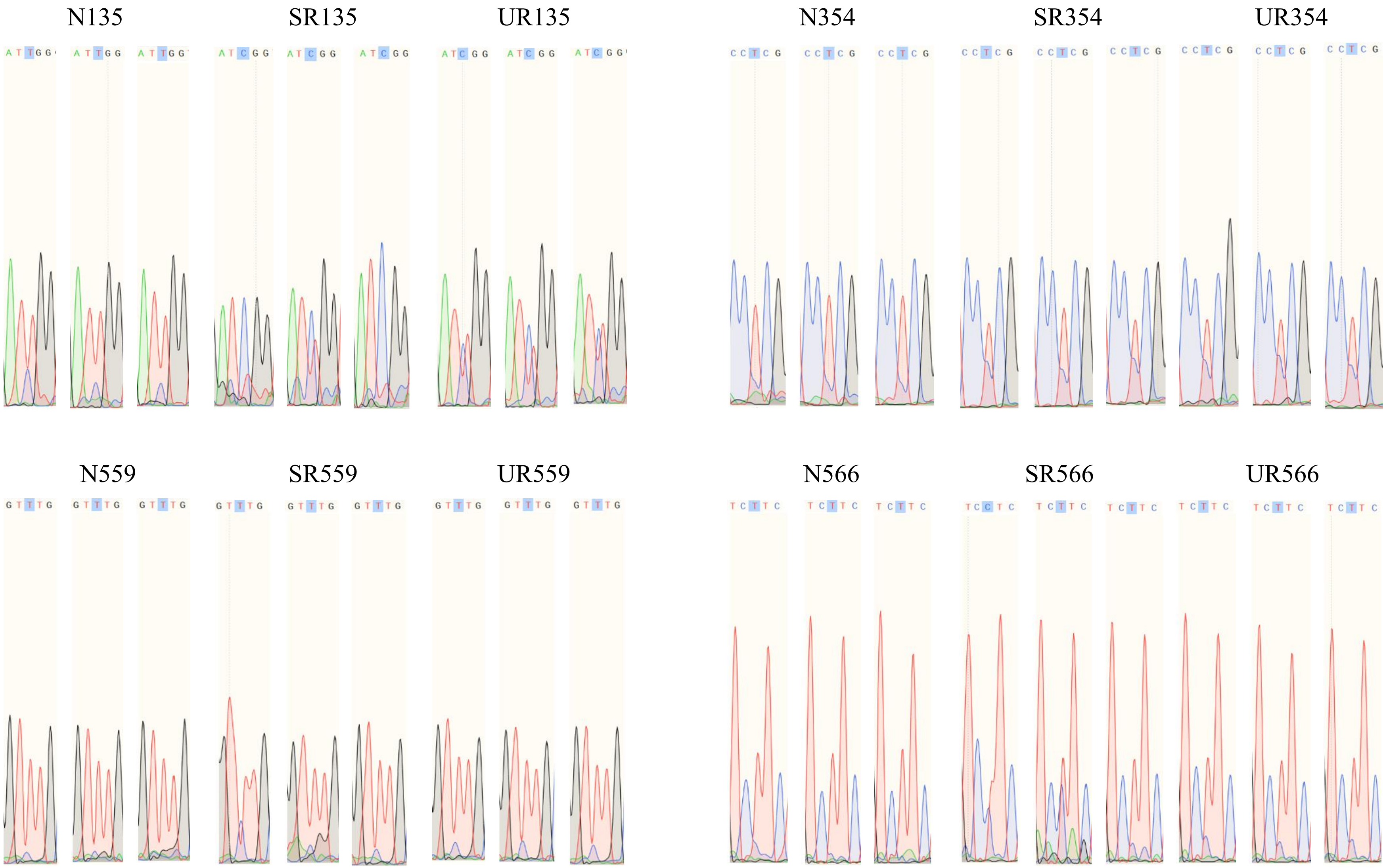

Supplementary Figure S1. Sanger sequencing results of three biological replicates of RNA editing sites on *matR* transcripts.

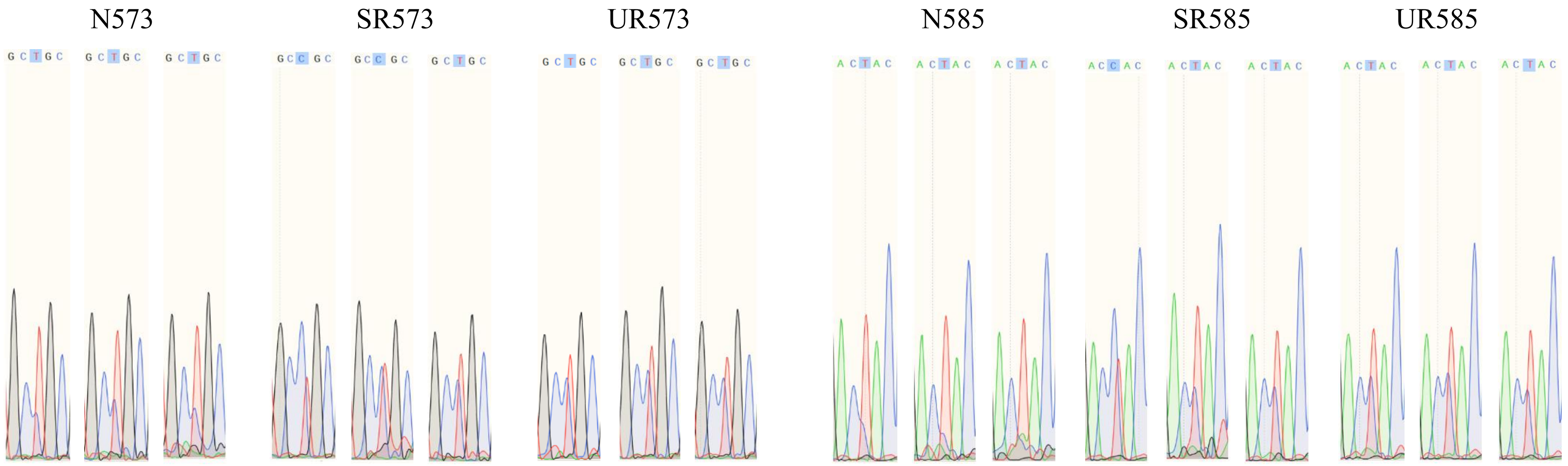

Supplementary Figure S1. Sanger sequencing results of three biological replicates of RNA editing sites on *matR* transcripts.
